# Bundibugyo virus disease and the unfinished countermeasure agenda

**DOI:** 10.2471/BLT.26.296654

**Published:** 2026-10-01

**Authors:** Nicola Abrescia, Antonio Capponi, Adelaide Maddaloni, Gabriella Molinaro, Maurizio D’Abbraccio

**Affiliations:** a AORN Dei Colli, D Cotugno Hospital for Infectious Diseases, Via Gaetano Quagliariello 54, 80131, Napoli, Italy.; b Faculty of Veterinary Medicine, University of Naples Federico II, Naples, Italy.; c Faculty of Medicine and Surgery, University of Naples Federico II, Naples, Italy.

Preparedness for Ebola disease has improved, but progress has been uneven across viral species and preparedness domains. Rapid case detection, molecular confirmation, infection prevention and control, contact tracing, supportive care, safe burial, community engagement and, for disease caused by the Zaire ebolavirus, vaccination and monoclonal-antibody treatment have changed outbreak management.^1^ Yet this progress has not produced comparable readiness for other clinically important orthoebolaviruses. Furthermore, preparedness can become a reductive crisis management effort when detached from the ecological, social and political conditions in which the outbreak occurs.^2^

The limited preparedness for Bundibugyo virus disease exposes both gaps. Identified in Uganda in 2007–2008, the virus recurred in the Democratic Republic of the Congo in 2012, and caused a 2026 outbreak there and in Uganda.^3^^–^^5^ Nearly two decades after its identification, candidate vaccines and therapeutics remained under evaluation, and outbreak-ready diagnostic pathways were still being expanded rather than being available as established tools at the outbreak’s onset.^6^^,^^7^

The World Health Organization (WHO) is evaluating candidate vaccines and therapeutics and has listed a molecular diagnostic test for Bundibugyo virus.^6^^,^^7^ However, listing an assay does not guarantee timely testing at peripheral or insecure sites. Anticipatory readiness requires investigational products, trial protocols, laboratory pathways, regulatory arrangements, procurement channels and community engagement to be in place before transmission accelerates. 

Preparedness should also apply to interfaces between animals, human and food sources. Here, risk-based veterinary intelligence refers to conducting targeted virological and epidemiological surveillance of live mammals, carcasses and animal products associated with hunting, trade, slaughter or consumption, integrated with human syndromic surveillance, case investigation and community reporting. However, natural infection and maintenance of Bundibugyo virus have not been confirmed in wild or domestic animal populations, no animal species has been implicated in the 2026 outbreak and the role of domestic animals remains uncertain.^8^ This uncertainty supports selective investigation guided by epidemiological signals rather than indiscriminate sampling, to improve interpretability, conserve resources and avoid unsupported attribution of risk to specific animals, foods or communities.

Surveillance at animal–human–food interfaces should address defined epidemiological questions. Priority signals include unusual illness or mortality in mammals used as food, animal exposure among human cases, carcass handling, informal slaughter, relevant markets, cross-border routes and potentially contaminated animal products. Samples should be collected only when safe investigation, referral, testing and communication pathways are available. In resource-constrained settings, low-yield animal sampling may displace investment in case management, infection prevention, contact tracing or routine services.

Evidence for the benefits of surveillance exists but should not be overstated. In Republic of the Congo and Gabon in 2001–2003, wild-animal mortality monitoring detected infected great-ape and duiker carcasses before human outbreaks, alerting health authorities to imminent risk on two occasions.^9^ The clearest value of this monitoring was early warning and epidemiological orientation: connected animal-health, laboratory and human-response systems could trigger targeted investigation and risk communication. However, the system did not prevent all transmission and did not prove that animal surveillance alone predicted or stopped human outbreaks. Rather, this evidence supports risk-based surveillance as an early-warning component within a broader response system.

The prevention and control trade-offs are substantial. Bushmeat, informal slaughter and open-air markets can be sources of exposure but are part of food security, income and local culture. Abrupt bans without compensation, alternative food sources or livelihood support may displace trade into less visible channels, reduce cooperation and undermine trust.^10^ Poorly targeted restrictions can stigmatize communities without reducing transmission. Community-centred response must therefore be operational: affected populations need clear information, negotiated restrictions, compensation when products are removed and protection from stigma.^11^

For Bundibugyo virus disease, animal-interface surveillance cannot substitute for isolation and care, infection prevention, contact tracing, safe burial or cross-border surveillance. Surveillance must be accompanied by adaptive clinical-trial capacity and equitable partnerships,^12^ and cannot replace sustained investment in local health and veterinary workforces, mobile laboratories, safe sample transport and essential services, especially in settings affected by conflict, displacement and mistrust. Surveillance can contribute to closing preparedness gaps where ecological uncertainty, food insecurity and human infection meet.

The lesson is not that Ebola countermeasures have failed, but that preparedness is too narrow when it focuses on disease caused by the Zaire ebolavirus, remains reactive and stays disconnected from the conditions that hinder spillover detection and transmission control. 
